# The Galvanic Effect of Titanium and Amalgam in the Oral Environment

**DOI:** 10.3390/ma13194425

**Published:** 2020-10-05

**Authors:** Patrick H. Carey IV, Shu-Min Hsu, Chaker Fares, George Kamenov, Fan Ren, Josephine Esquivel-Upshaw

**Affiliations:** 1 Department of Chemical Engineering, University of Florida, Gainesville, FL 32608, USA; careyph@ufl.edu (P.H.C.IV); c.fares@ufl.edu (C.F.); fren@che.ufl.edu (F.R.); 2 Department of Restorative Dental Sciences, Division of Prosthodontics, University of Florida College of Dentistry, Gainesville, FL 32608, USA; shuminhsu@ufl.edu; 3 Department of Geological Sciences, University of Florida, Gainesville, FL 32608, USA; kamenov@ufl.edu

**Keywords:** titanium, amalgam, ICP-MS, corrosion, galvanic, XPS

## Abstract

The effects of the presence of amalgam on titanium (Ti) dissolution in the oral environment under acidic, neutral, and basic conditions was studied. The presence of amalgam was found to suppress Ti release under acidic conditions due to the redeposition of TiO_x_/SnO_x_ on the surface of the Ti. The redeposition of SnO_x_ was due to the amalgam releasing its components (Hg, Cu, Sn, Ag) and the thermodynamic preference of Sn to oxidize, which was confirmed using mass measurements, ICP-MS analyses, and X-ray Photoelectron Spectroscopy (XPS). XPS depth profiling was performed to characterize the composition and oxidation states of the redeposited SnO_x_/TiO_x_ film. Under basic conditions, the amalgam hindered Ti dissolution, but no redeposition of amalgam components was observed for the Ti.

## 1. Introduction

The first titanium (Ti) dental implant was placed into Gösta Larsson by Dr P.I. Brânemark in 1965. The patient had four implants placed into his mandible; within 6 months, all were osseointegrated and remained within the patient for the next 40 years [1,2,3,4,5,6]. This was no fluke, as Dr. Brânemark, during his graduate studies, had observed the fusion of Ti wire to bone. With dental implant prostheses becoming the standard of care in many countries, the subtle issues with implant technology are becoming apparent. One of the issues is the placement of two dissimilar metals within a patient. This occurs quite commonly when a patient has an implant along with amalgam fillings. Amalgam has quite a storied history and has had a place in restorative dentistry since the early 1800s in England, although amalgam’s initial use was noted by the Chinese in 659 AD [7]. If amalgam begins to corrode either mechanically or chemically within a patient, there is a release generally of tin, copper, zinc, silver, and mercury [8,9,10,11]. While the released amounts may be miniscule, there is still a risk of exposure to heavy metals, especially for young children or women who are childbearing or breast feeding. As of 2004, the WHO prescribed a tolerable intake of 1.6 µg Hg/kg bodyweight for these at-risk groups [12]. Generally, this limit is reached through food-related absorption, as a person would need to have approximately 490 amalgam surfaces to release this quantity of mercury from their dental restorations alone [13].

The specific issue with the placement of two dissimilar metals within the oral environment is the difference in reduction potentials. A galvanic cell is created where saliva or tissue acts as a conductor between two dissimilar metals (electrodes). One of the two metals will be reduced, while the other will be oxidized. The loss of material into the surrounding tissue is a major concern, as this can lead to inflammation, the creation of a local acidic environment, oral microbiome changes (bacterial proliferation and/or suppression), or other side effects. In addition to the loss of material, the generation of a current between the two electrodes can lead to localized positive/negative charges on the implant surface that may hinder bone absorption [14,15,16].

There are numerous methods being undertaken to preserve titanium implant integrity, and these primarily involve coating with a metallic spice. Coating with a Ti-X alloy may be preferential, as the coating will adhere strongly to the underlying Ti [17,18,19,20,21,22,23]. Other coatings based on Ta, BN, Nb, NbO, NbN, NbC, hydroxyapatite, and CuO have also been explored [18,24,25,26,27,28]. The problem with any thin-film coating is the problematic screwing of the implant into the mandible, which can wear down and away the coating. Additionally, once the coating has been compromised, the impact on the implant lifetime is not yet well-understood for most films. Most current implants make use of a TiN coating which has been demonstrated to improve osseointegration and improve corrosion resistance [29,30,31,32,33,34,35,36]. However, no studies have observed a TiN coated when placed in the oral environment with another metal.

In this study, Ti cylinders were submerged in pH 2, pH 7, and pH 10 buffer solutions both individually and as a galvanic cell with amalgam. These pHs were selected to place the results on the extreme to identify predominant trends. The results were quantified by optical imaging, mass measurements, ICP-MS, and x-ray photoelectron spectroscopy (XPS). The aim of this research is to determine whether significant amounts of Ti are released into the oral environment and whether the creation of a galvanic cell with amalgam will have significant effects on the amount of Ti released.

## 2. Materials and Methods

**Figure 1:**
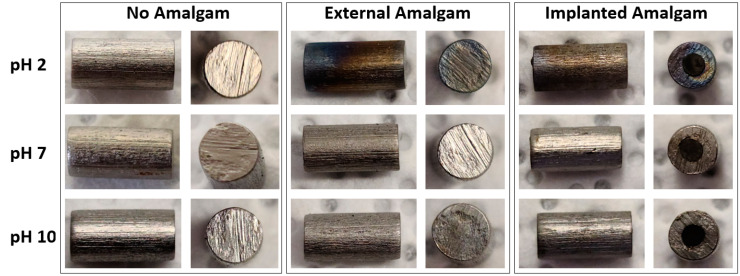
Optical images of Ti rods immersed in pH 2, pH 7, and pH 10 solution.

Ti rods (1/4” Diameter × 1/2” Length) purchased from Kurt J. Lesker (Jefferson Hills, PA, USA) of 99.995% purity were used for the experiment. The amalgam was purchased from Tytin (regular set, high Cu) (Brea, CA, USA). The self-activating capsules containing 59% Ag, 28% Sn, and 13% Cu were mixed with mercury according to manufacturer’s instruction to yield final weight percentages of 42.5% Hg, 33.9% Ag, 16.1% Sn, and 7.5% Cu. The experiment used 3 experimental groups: Ti rods without amalgam, Ti rods with implanted amalgam, and Ti rods with external amalgam that was not in direct contact with the rod (see Figure 1 for images). The various pH solutions were commercially purchased from Fisher Scientific (Waltham, MA, USA). The rods were individually submerged in 15 mL of each pH buffer in new polypropylene centrifuge tubes and maintained at 37 °C in a shaken water bath for a total of 4 weeks. After 2 weeks, the rods were removed, rinsed with deionized water, and allowed to dry overnight in a desiccator. Their mass was taken 3 times, and the result was averaged. The rods were then placed in a new buffer solution with new tubes for an additional 2 weeks. The samples underwent the same cleaning and mass measurements at the 4 week mark. The individual used buffer solutions at the 2 week mark were used in ICP-MS analyses. The raw count per second data may be found in the supplementary material supplementary material Tables S1–S5.

Mass measurements were performed using a Radwag (Miami, FL, USA) AS60/220.RX Analytical Balance with an error of ±0.015 mg.

The pH 10 buffer solution contained 97.5% water, 1% ethylenediaminetetraacetic acid, disodium salt dihydrate, 0.6% potassium carbonate, 0.5% potassium hydroxide, and 0.4% potassium borate. The pH 7 buffer solution contained >98% water, 1.47% sodium phosphate dibasic, and 0.35% dihydrogen potassium phosphate. The pH 2 buffer solution contained 99.43% water, 0.4% potassium chloride, 0.1% hydrochloric acid, 0.05% formaldehyde, and 0.02% methyl alcohol. 

A small aliquot of the buffer solution after the experiment was removed and diluted with an additional 0.8 N HNO_3_, spiked with 8 ppb Rh and Re, so that the final dilution was around 25× The final dilution for the trace element analyses was determined by weight for each sample. Trace element analyses were performed on a ThermoFisher Scientific Element 2 HR-ICP-MS (Waltham, MA, USA) with a medium resolution, with Re and Rh used as internal standards. The quantification of results was performed by external calibration using gravimetrically prepared Cu, Ag, Sn, and Ti standards from stock ICP-MS solution standards. The reported concentration values for Cu, Ag, Sn, and Ti are better than +/−5%. In addition to the above four metals, we also attempted to measure the Hg. However, due to high Hg memory effect in the introduction system of the ICP-MS, the determination of Hg concentrations in the buffer sample solutions was not successful. 

The X-ray Photoelectron Spectroscopy (XPS) system was a Physical Instruments ULVAC PHI (Chanhassen, MN, USA) with a monochromatic Al x-ray source. The source power was 300 W, with an energy of 1486.6 eV, a takeoff angle of 50°, an acceptance angle of 7°, and an analysis area 100 µm in diameter. The electron pass-through energy was 93.5 eV for survey scans and 23.5 eV for high-resolution peak scans. Ar sputtering was used to depth profile the samples at an energy of 2 kV. The adventitious C1s peak at 284.4 eV was used to align the spectra. The Shirley background was subtracted from each peak region and each peak was fitted with a Gaussian distribution.

## 3. Results and Discussion

There has been a lack of literature which quantifies the amount of metallic release during this occurrence. Lim et al. (2002) performed the first study using Inductively Coupled Plasma-Mass Spectrometry (ICP-MS). Lim focused primarily on the dissolution of amalgam and the surface roughening that occurred to the amalgam, and did not provide significant discussion of the Ti changes [37]. This study was performed in a NaCl solution, likely at a near-neutral pH. The next closest study was by Bajsman et al. in 2014, quantifying the amalgam release in synthetic saliva via ICP-MS. In this study, a galvanic cell was not formed with Ti, but the electrolytic solution used was synthetic saliva (pH 6.5) in line with the formulation by Duffo [38]. As such, there is a definite deficit in the literature which provides a discussion of the effects of the oral environment on a Ti-amalgam galvanic cell. The oral environment has a fluctuating pH usually closer to 5.5, but can range from pH 2 to 10 depending on food and drink choices. For this experiment, we aim to begin the clarification of the effects of pH and the galvanic cell on Ti corrosion.

### 3.1. Reduction Potential

Before the experimental results can be discussed, establishing what reactions are expected to occur is important. Several key reduction potentials for each metal are shown in Table 1.

The standard cell potential for a galvanic cell is a positive value, so the reaction at the anode will be a half reaction, with a smaller or more negative standard reduction potential. By first reversing the half reaction of *Ti* and then adding that equation to the half reaction of *Hg*^2+^, we find the following:
$Ti⇌{Ti}^{2+}+2e^{-}$+1.63 V$+Hg^{2+}+2e^{-}⇌Hg$+0.85 V$Ti+Hg^{2+}⇌Hg+Ti^{2+}$+2.48 V

This calculation can be performed for the other metallic species in amalgam, as all have less negative reduction potentials than *Ti* and give the same result—that *Ti* will be oxidized. 

### 3.2. Optical Observation

Optical images are presented for three sets of Ti rods in Figure 1. The first set was placed in the various pH solutions without amalgam, and even after 4 weeks submersion the rods demonstrated no observable change. Two sets of rods with amalgam were submerged. One set placed the amalgam in intimate contact by drilling out the center of the rod and then implanting the amalgam inside the drilled hole. This scenario of intimate contact between the Ti and amalgam would not occur in practice; this data set was used to demonstrate the extreme scenario of the closest possible contact of amalgam and Ti. The other set placed the amalgam external to the rod but in the same solution. The two sets with amalgam demonstrated similar observable effects, where under acidic conditions a noticeable color gradient was visible on the Ti rod. This color change would be indicative of the formation of a thin film on the Ti rod, and the gradient of color would be due to variation in the thin-film thickness. This film growth or oxidation is consistent with the reduction potentials of all the metals present. The rods with amalgam in basic conditions also exhibited minute amounts of discoloration, with a slight shade of brown appearing on some areas of the rod.

To quantify the amount of Ti lost, the rods’ mass was taken at the 2- and 4-week mark of submersion at 37 °C (Table 2). The rods without amalgam and the rods with external amalgam demonstrated no detectable change in mass. Only the rods with implanted amalgam showed a loss of mass; however, this is likely due to the weight loss of the implanted amalgam, as the rods with external amalgam showed no weight loss. This cannot be directly measured, as the implanted amalgam cannot be removed from these samples. Amalgam has previously been shown to release significant quantities of elements into the surrounding environment even in neutral solutions [37]. The result of this mass loss experiment demonstrated that the Ti loss was below the limit of detection (LOD) for the scale used (10^−5^ g).

### 3.3. Supernatant Analysis

To better quantify the Ti release, ICP-MS analyses were performed for the various solutions used to soak the rods and amalgam. To establish proper baselines, ICP-MS analyses were performed on the new buffer solutions prior to the introduction of the Ti and amalgam. The results are presented in Table 3, where all the values are better than +/−5%. Table 3 indicates that there is a small amount of Ti, Cu, Ag, and Sn contamination in all of the buffers. However, the presence of metal impurities in the stock buffers is relatively minor when compared to the release of each ion, as discussed later in the manuscript.

In Table 4, the ICP-MS results for Ti rods without amalgam are presented. Under both acidic and basic conditions, there is evident Ti release. Under neutral conditions, no release is noted, as the measurement is comparable to that of the original buffer solution in Table 3. The acidic condition result was expected, as Ti is released when converted from Ti to Ti^2+^ and Ti^4+^. The dissolution in basic solution comes as a relative surprise, as an oxide film would be expected to form, hindering dissolution. Mentus et al. studied the corrosion of Ti in 1–5M of NaOH and saw a similarly surprising result [40]. What may be occurring is the formation of low-quality TiO_2_, with the subsequent dissolution of TiO_2_ by the production of titanates:

$${\mathrm{TiO}}_{2}+2\mathrm{KOH}⇌K_{2}{\mathrm{TiO}}_{3}+H_{2}O$$

Normally, these types of processes must occur at very high temperatures >850 °C [41]. However, this work is similar to the result obtained by Mentus et al., where there may be interrelated aging effects that cause the film to dissolve more easily, as the submersion for this experiment lasted for 4 weeks. There is a minor increase in the Cu, Ag, and Sn measurements in the solutions after the experiments (Table 4). The small presence of these ions may be due to minor sample contamination during the experiment. The quantity of these ions is relatively minor and does not present a challenge to the interpretation of Ti dissolution.

It is important to now remember our previous discussion of the reduction potentials. When Ti is present with the components of amalgam, Ti will be oxidized rather than reduced. In Table 5, the ICP-MS results of the Ti rods with external amalgam are presented. Under acidic conditions, the Ti is no longer released, as the Ti is now oxidized (the amalgam is reduced) due to the presence of the amalgam, halting Ti dissolution. Under a basic pH, Ti dissolution occurs, but not to the same extent as in Table 4, due to the stronger oxidation from the galvanic cell (higher-quality oxide formation). The Sn measurements show a stark difference from acidic to basic conditions. This discrepancy arises due to Sn redeposition under acidic conditions on the titanium surface where Ti is oxidized, meaning that Sn is no longer in the solution. This result will be discussed in the following XPS results and Gibbs free-energy calculations. Cu and Sn were observed to be released under both conditions (Table 5). Small amounts of Ag were demonstrated in the test solutions, which may have arisen from the precipitation of AgCl_2_ in the test solutions where the supernatant was tested. A significant amount of Hg was also present in these solutions; however, the first amalgam solution contaminated the ICP-MS introduction system. This created a major Hg memory effect in the ICP-MS, and the quantification of Hg was not possible (see Appendix for raw cps data to demonstrate the memory effect). Regardless of the memory effect, the observed high Hg intensities in the amalgam solutions provide qualitative evidence for significant Hg dissolution, as expected from the galvanic cell reaction. This is further confirmed by the mass loss reported in Table 2.

### 3.4. Surface Characterization

**Figure 2:**
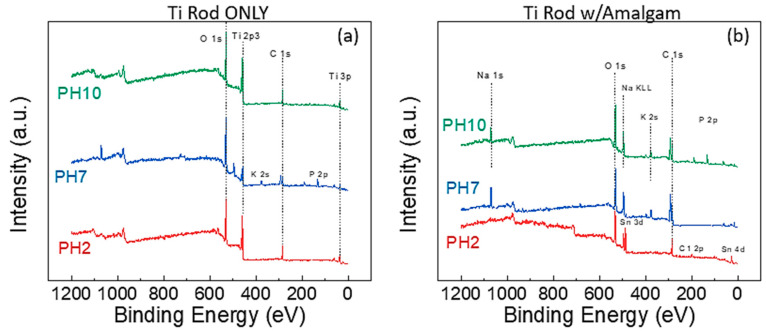
XPS survey spectra of (**a**) Ti rods submerged in various solutions without amalgam and (**b**) Ti rods submerged in various solutions with amalgam.

The next step was to establish what chemical changes were occurring at the surface of Ti, as a physical film growth was observed for the rods at pH 2 when a galvanic cell was present. The rods placed in the solution without amalgam all demonstrated essentially the same surface chemistry (Figure 2a), as the same XPS peaks were present in all the samples: O1s, Ti 2p3, C1s, and Ti 3p. Minor peaks for K and Na were noted in some of the samples, as these ions were present in the buffer solutions. The samples were cleaned with deionized (DI) water prior to XPS to remove these unbound ions/salt, but apparently some K and Na remained on the Ti rods. 

Figure 2b presents the survey scans for the Ti rods with external amalgam in the various solutions. The immediate difference is that the Ti 2p3 peak is not visible on any of the spectra; indicating a significantly thicker oxide layer along with the substantial presence of sodium from the buffer solution. The pH 2 additionally shows a significant presence of a Sn 3d peak. The Sn peak was of further interest, as this peak would indicate the redeposition of the Sn from the corroded amalgam.

**Figure 3:**
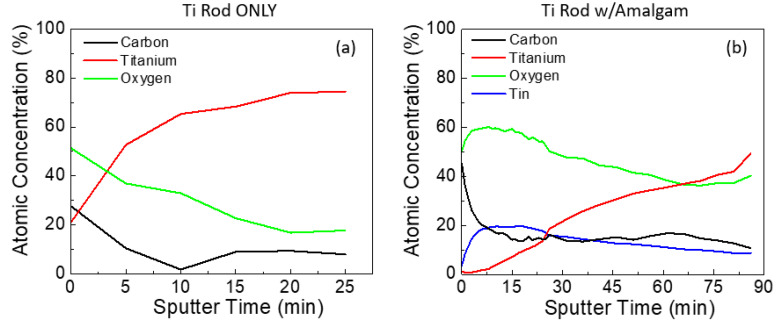
XPS depth profiles of the Ti rod immersed at pH 2 (**a**) without amalgam and (**b**) with amalgam.

XPS depth profiling was performed on samples both with and without amalgam at pH 2 to provide a comparison for the thickness of this observable film. One of the challenges with sputter depth profiling is that the rate is dependent on the specie being sputtered. Thus, as the composition changes, the sputter rate also changes. For this reason, we present the sputter depth profile results with respect to time and not depth. For the rod without amalgam, the TiO_x_ film takes approximately 20 min at 2 kV to sputter through before a constant composition is reached (Figure 3a). For the rod with amalgam, even after nearly 90 min of sputtering the entirety of the SnO_x_ film was not removed, indicating that this layer is very thick and may be in the order of microns thick (Figure 3b). 

**Figure 4:**
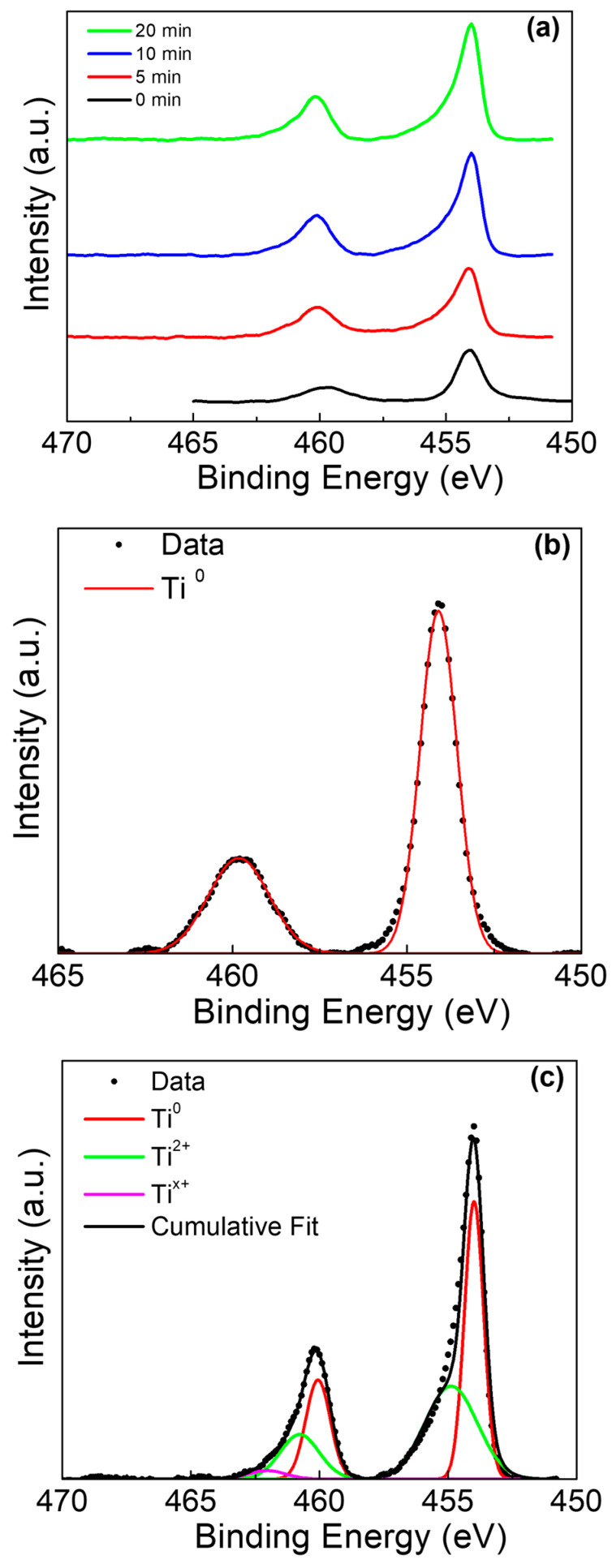
Ti 2p XPS of Ti rod without amalgam in pH 2. (**a**) Ti 2p peak at various sputter times, (**b**) deconvolution of 0 min sputter time, and (**c**) deconvolution of 20 min sputter time.

Peak deconvolution was performed on the Ti 2p, Sn 3d, and O 1s peaks for both Ti rods with and without amalgam placed in acidic solutions. Beginning with the Ti rods without amalgam, the stacked overlay of the Ti 2p peaks is presented at multiple sputtering times in Figure 4a. The analysis of the Ti 2p peak observed the spin orbit doublet peaks at ~454 eV (Ti^0^ 2p_1/2_) and ~459.8 eV (Ti^0^ 2p_3/2_) at the surface of the sample in Figure 4b (0 min), which is consistent with previous reports on TiO_2_ surfaces [42,43]. As the sputtering of the surface continued, the presence of the Ti^2+^ peaks became apparent at 455.3 eV (Ti^2+^ 2p_3/2_) and 460.8 (Ti^2+^ 2p_1/2_), as shouldering occurred in the primary metallic peaks [44]. A third peak was identified at ~461.8 eV, which has been observed in samples with intermixed and poorly defined Ti^2+^ and Ti^3+^; this third peak is likely a non-stoichiometric titanium oxide, and this has been labelled as Ti^x+^ [45].

**Figure 5:**
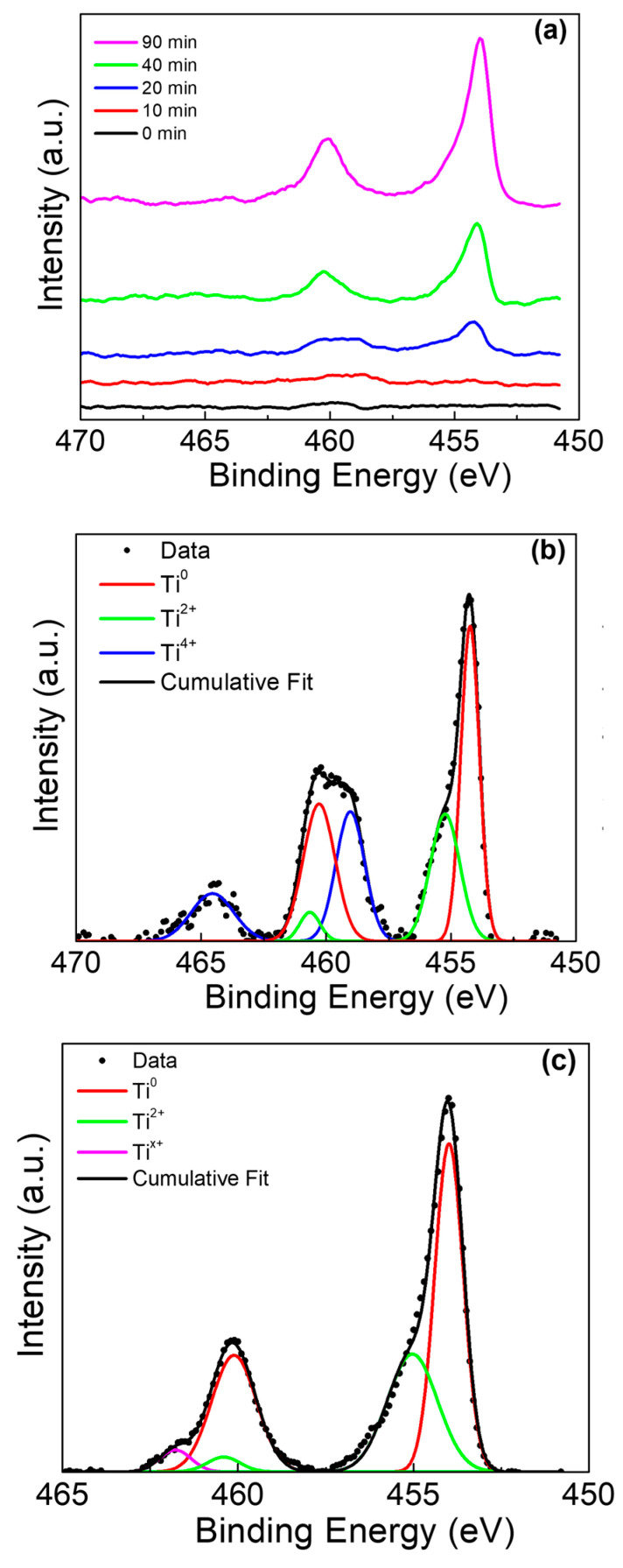
Ti 2p XPS of Ti rod with amalgam at pH 2. (**a**) Ti 2p peak at various sputter times, (**b**) deconvolution of 20 min sputter time, and (**c**) deconvolution of 90 min sputter time.

The Ti rod with amalgam presented a more interesting case, as initially Ti was not observed on the sample surface by XPS. The stacked overlay shows the evolution of these peaks as the sample is sputtered (Figure 5a). Peak deconvolution was performed after sputtering for 20 min (Figure 5b), and after 90 min (Figure 5c). The presence of a broad peak at ~460 eV was determined to be a mixed composition of Ti^0^, Ti^2+^, and Ti^4+^. As sputtering continued, Ti^4+^ was no longer observed and a weak Ti^x+^ peak became present, mirroring the result in Figure 4c. 

**Figure 6:**
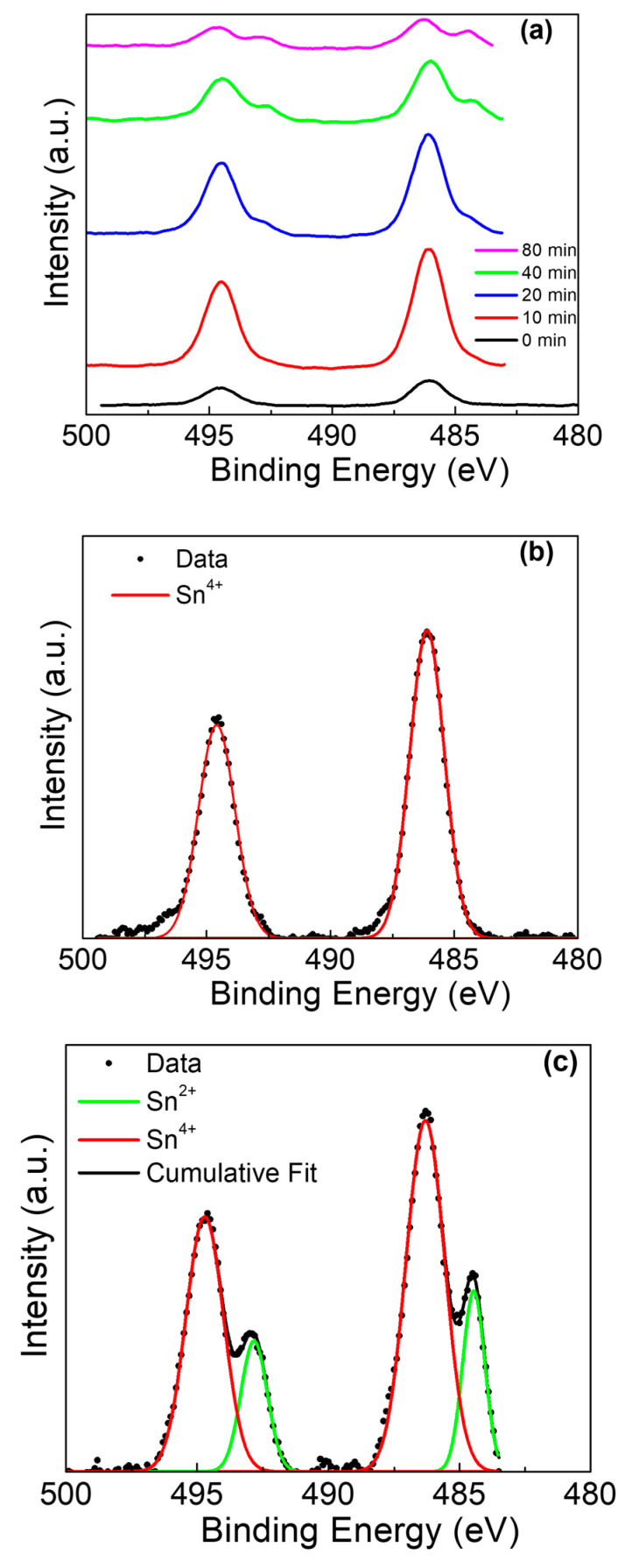
Sn 3d XPS of Ti rod with amalgam at pH 2. (**a**) Sn 3d peak at various sputter times, (**b**) deconvolution of 0 min sputter time, and (**c**) deconvolution of 90 min sputter time.

While the Ti presented a depth-dependent phase evolution, the Sn also presented significant changes with depth (Figure 6a). At the surface (Figure 6b), the Sn 3d exhibited spin-orbit doublet peaks at 486.1 eV(Sn^4+^ 3d_5/2_) and 494.6 eV (Sn^4+^ 3d_3/2_), with a peak separation of 8.5 eV [45,46,47]. As the sputtering progressed, the mixed tin-titanium oxide layer was revealed and the presence of Sn^2+^ became strongly apparent (Figure 6c), with spin doublet peaks at 492.8 eV (Sn^2+^ 3d_3/2_) and 484.4 eV (Sn^2+^ 3d_5/2_) and a peak separation of 8.4 eV.

**Figure 7:**
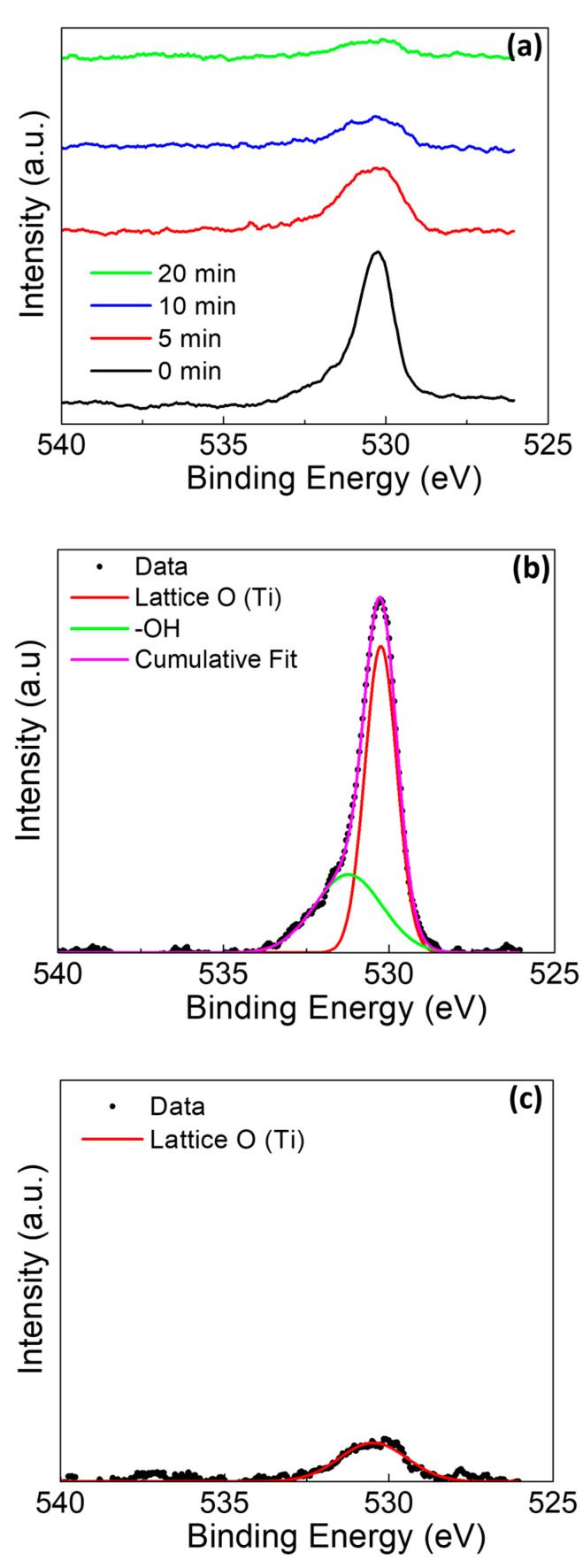
O1s XPS of Ti rod without amalgam at pH 2 (**a**) at various sputter times, (**b**) at deconvolution of 0 min sputter time, and (**c**) at deconvolution of 20 min sputter time.

The O1s peak for the rod without amalgam (Figure 7) demonstrated a depth-dependent relative intensity of the lattice site oxygen (530.5 eV) and terminating/bridging hydroxyl groups (531.3 eV) [24,43,48]. As the sputter profile continues, the hydroxyl peak intensity disappears as the bulk of the sample is reached. 

**Figure 8:**
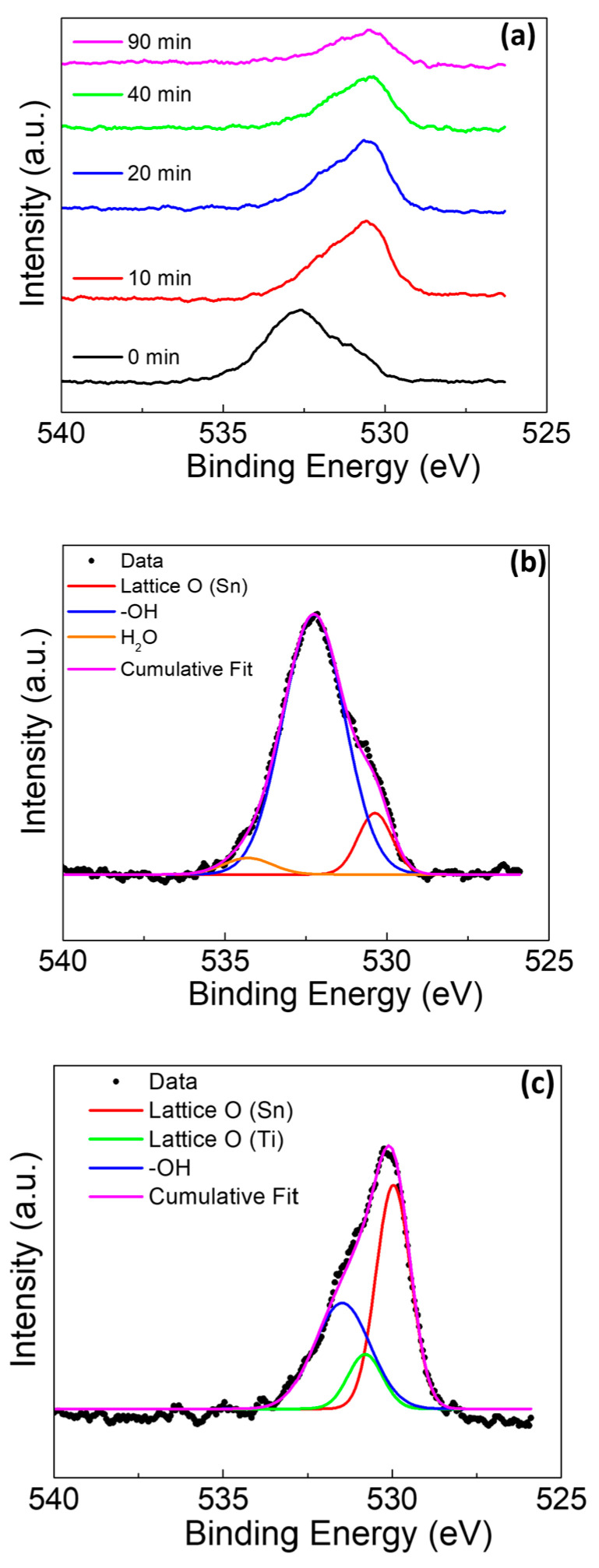
O 1s XPS of Ti rod with amalgam at pH 2 (**a**) at various sputter times, (**b**) at deconvolution of 0 min sputter time, and (**c**) at deconvolution of 20 min sputter time.

In Figure 8, the O1s spectra for the sample with amalgam again presented with several unique characteristics as compared to the sample without amalgam. The first characteristic in Figure 8a is that the surface appears to be heavily terminated with -OH groups, with only a weak presence of lattice site oxygen associated with Sn at 530.1 eV [45,46]. There was no observed peak for the lattice site oxygen associated with Ti. There was slight shouldering due to the presence of H_2_O at 534.3 eV [48]. The H_2_O may be adsorbed to the surface or incorporated into a rapidly grown mixed oxide film. As the depth profiling continued, the shouldering associated with H_2_O was not noted (Figure 8a). As the profiling continued, the peak for the lattice oxygen (Ti) presence became evident at 530.7 eV (Figure 8c) [42,43,48]. The minor shouldering for water was not observed with the sputtering, likely indicating that the water observed at the surface is adsorbed rather than incorporated into the film, as water’s continued presence would indicate the incorporation into the film. 

### 3.5. Thermodynamic Principles for SnO_x_ Regrowth

The last major consideration is why Sn was the only element seen to redeposit as an oxide film and why Ag, Cu, and Hg did not. To understand this phenomenon, turning to rudimentary thermodynamic principles is necessary. Table 6 presents the enthalpy of the formation and entropy of key species.

For all of the released metal ions, we consider the two most likely outcomes, the formation of an oxide or chloride, and in the simplest reaction pathways (Table 7). This analysis will not consider the chlorine exchange reactions between ions. The Gibbs free energy was calculated for each reaction pathway using the values present in Table 6. Gibbs free energy values are useful in determining whether a reaction will happen spontaneously and under what temperature conditions. For this case, we only consider the reaction at 37 °C. For negative values of Gibbs free energy, a reaction is considered to happen spontaneously. The key observation is that Hg and Ag will spontaneously form chlorides, while Cu and Sn will spontaneously form oxides. For Cu, the difference in spontaneity to form an oxide versus chloride is small, at only 9 kJ/mol, favoring the oxide; considering the small composition of Cu in the amalgam, Cu levels may have been below the detection limits of the XPS system. Sn, on the other hand, has an extremely large difference of 514 kJ/mol, favoring the oxide, and is present to a much greater amount in amalgam. The redeposition of Sn at the Ti electrode is necessary, as the Sn, once reduced to Sn^4+^ in solution, will not easily oxidize in the acidic solution. Due to the galvanic cell, oxidation only occurs at the Ti electrode, and as such the Sn^4+^ ions will plate on the Ti in order to oxidize.

Under the basic conditions, with the presence of -OH ions in solution the Sn does not need to be redeposited on the surface of the titanium in order to oxidize, as it can do so freely in solution.

Further work must be undertaken to determine to what extent the results of this work are observed in live subjects. Namely, the Ti and amalgam release should be quantified in the surrounding tissue and so should their effect on the host. Additionally, the formation of the mixed Sn/Ti oxide may lead to unanticipated changes in the osseointegration of the implant. In a live environment, many new interactions will be introduced that may enhance and/or confound this manuscript’s results, such as the natural fluctuation of pH in the mouth, oral bacteria, oral hygiene, etc.

## 4. Conclusions

In this study, the corrosion of Ti both individually and in a galvanic cell with amalgam was investigated under acidic, neutral, and basic conditions. Ti individually was observed to corrode under both acidic and basic conditions. The amalgam’s presence suppressed Ti corrosion under acidic conditions due to the redeposition of SnO_x_ on the surface of the Ti; all other ions were not observed to redeposit. Gibbs free energy, which relates to the change in the enthalpy and entropy of a given reaction to determine whether a reaction will be spontaneous, can be used to explain this phenomenon. In this case, Ag and Hg both presented a preference for forming a chloride in solution. Cu presented a weak (~11 kJ/mol) preference for oxide formation, but both chloride and oxide could form spontaneously (ΔG < 0), and, given the acidic condition, chloride ions were readily present in the solution. Sn presented a large (514 kJ/mol) preference for oxide formation, and the chloride cannot form spontaneously (ΔG > 0). Due to these thermodynamic preferences, the Sn ions in solution would oxidize on the titanium surface, as the titanium is oxidized while the amalgam is reduced due to the reduction potentials of each metal. XPS depth profiling was used to extract information on the phase change in Sn and Ti through this redeposited layer. This result challenges previous assumptions that Ti is entirely bioinert and does not corrode due to the formation of a passivating oxide film on the surface.

## Figures and Tables

**Table 1:** Reduction potentials for *Ti*, mercury, silver, tin, and copper at 25 °C [39].

Reaction	E_0_ (V)
$Ti^{2+}+2e^{-}⇌Ti$	−1.63
$Hg^{2+}+2e^{-}⇌Hg$	0.85
$Ag^{+}+e^{-}⇌Ag$	0.80
$Sn^{2+}+2e^{-}⇌Sn$	−0.14
$Cu^{2+}+2e^{-}⇌Cu$	0.34

**Table 2:** Mass loss of the Ti rods without amalgam, Ti rods with external amalgam, and implanted amalgam after 2 weeks and 4 weeks of soaking in a buffer solution at 37 °C. (Note: <LOD = a mass measurement below the limit of detection of the scale used.)

Sample	pH	Mass Loss Week 0 to Week 2 (µg/cm^2^ (Ti)/ cm^2^ (Amalgam)/Day)	Mass Loss Week 2 to Week 4 (µg/cm^2^ (Ti)/ cm^2^ (Amalgam)/Day)
Ti	2	<LOD	<LOD
Ti	7	<LOD	<LOD
Ti	10	<LOD	<LOD
Ti + External Amalgam	2	<LOD	<LOD
Ti + External Amalgam	7	<LOD	<LOD
Ti + External Amalgam	10	<LOD	<LOD
Ti + Implanted Amalgam	2	1849 ± 383	975 ± 175
Ti + Implanted Amalgam	7	438 ± 315	0 ± 145
Ti + Implanted Amalgam	10	2846 ± 657	582 ± 218

**Table 3:** ICP-MS analyses of the new buffer solutions and limits of detection (LODs).

Ion	pH 2 Buffer	pH 7 Buffer	pH 10 Buffer	LOD
Ti (ppb)	0.68	6.76	4.37	0.02
Cu (ppb)	0.19	0.18	2.52	0.01
Ag (ppb)	0.07	0.05	0.05	0.005
Sn (ppb)	0.78	1.18	0.77	0.02

**Table 4:** ICP-MS analyses of solution containing submerged Ti rods at pH 2, 7, and 10.

Ion	Rod in pH 2	Rod in pH 7	Rod in pH 10
Ti (ppb)	165	8.08	142
Cu (ppb)	6.37	2.74	5.35
Ag (ppb)	10.0	0.07	0.09
Sn (ppb)	8.83	4.82	3.64

**Table 5:** ICP-MS of solution containing submerged Ti rods with external amalgam at pH 2, 7, and 10.

Ion	pH 2	pH 7	pH 10
Ti (ppb)	0.58	6.87	66.3
Cu (ppb)	2.7 × 10^5^	35	6.0 × 10^5^
Ag (ppb)	5.67	0.79	17.1
Sn (ppb)	58.6	112	5.8 × 10^5^

**Table 6:** Enthalpy of the formation and entropy values for key species.

Formula	$\Delta H_{f}^{}\;(\mathrm{kJ}/\mathrm{mol})$	S (J/Kmol)
$Ag^{+}$ *(aq)*	106	72.7
$Ag_{2}O$ *(s)*	−31	122
AgCl *(s)*	−127	96.2
$Cl^{-}\;\left(aq\right)$	−167	56.5
$Cu^{+}$ *(aq)*	72	40.1
$Cu_{2}O\;$ *(s)*	−169	93.1
CuCl *(s)*	−139	87.1
$H_{2}\;\left(g\right)$	0	131
$H_{2}O\;\left(l\right)$	−285	70.0
$Hg^{2+}\left(aq\right)$	172	−32.2
HgO (s)	−91	70.3
$HgCl_{2}\;\left(s\right)$	−230	145
$Sn^{4+}\;\left(aq\right)$	30.5	−117
$SnO_{2}\;\left(s\right)$	−577	52.3
$SnCl_{4}\;\left(s\right)$	−529	300

**Table 7:** Thermodynamic calculations for the formation of key oxides and chlorides.

Product	Reaction	$\Delta H_{f}\;(\mathrm{kJ}/\mathrm{mol})$	ΔS (J/K mol)	$\Delta G_{f}\;(\mathrm{kJ}/\mathrm{mol})$
$Ag_{2}O\;$(s)	$2Ag^{+}\left(aq\right)+H_{2}O\left(l\right)⇌Ag_{2}O\left(s\right)+H_{2}$	42	38	31
AgCl (s)	$Ag^{+}\left(aq\right)+Cl^{-}\left(aq\right)⇌AgCl\;$(s)	–66	–33	–56
$Cu_{2}O$(s)	$2Cu^{+}\left(aq\right)+H_{2}O\left(l\right)⇌Cu_{2}O\;\left(s\right)+H_{2}\;\left(g\right)$	–28	74	–50
CuCl (s)	$Cu^{+}\left(aq\right)+Cl^{-}\left(aq\right)⇌CuCl\;\left(s\right)$	–44	–10	–41
HgO (s)	$Hg^{2+}\left(aq\right)+H_{2}O\left(l\right)⇌HgO\left(s\right)+H_{2}\left(g\right)$	22	164	–28
$HgCl_{2}\;\left(s\right)$	$Hg^{2+}\left(aq\right)+2Cl^{-}\left(aq\right)⇌HgCl_{2}\left(s\right)$	–68	64	–88
$SnO_{2}\;\left(s\right)$	$Sn^{4+}\left(aq\right)+H_{2}O\left(l\right)⇌SnO_{2}\left(s\right)$	–38	291	–463
$SnCl_{2}\;\left(s\right)$	$Sn^{4+}\left(aq\right)+4Cl^{-}⇌SnCl_{4}\left(l\right)$	109	191	51

## Supplementary Materials

The following are available online at https://www.mdpi.com/1996-1944/13/19/4425/s1. Table S1: Titanium calibration intensity avg, Table S2: Blank test solutions used in experiment intensity avg, Table S3: Solutions from soaking Ti rods only (no amalgam) intensity avg, Table S4: Solutions from soaking Ti rods with amalgam intensity avg, Table S5: Final wash of ICP-MS to demonstrate the memory effect of Hg.
