# Supplementary material for: The Galvanic Effect of Titanium and Amalgam in the Oral Environment
Source: Materials (Basel). 2020 Oct 5;13(19):4425. doi: 10.3390/ma13194425 (PMC7579048; doi:10.3390/ma13194425)
Supplement: Supplementary file 1 [file materials-13-04425-s001.pdf]

Supplementary Materials

The Galvanic Effect of Titanium and Amalgam in the Oral Environment

Patrick H. Carey IV [1], Shu-Min Hsu [2], Chaker Fares [1], George Kamenov [3], Fan Ren [1] and Josephine Esquivel-Upshaw *,[2]

[1] Department of Chemical Engineering, University of Florida, Gainesville, FL 32608, USA; careyph@ufl.edu (P.C.); c.fares@ufl.edu (C.F.); fren@che.ufl.edu (F.R.)
[2] Department of Restorative Dental Sciences, Division of Prosthodontics, University of Florida College of Dentistry, Gainesville, FL 32608, USA; shuminhsu@ufl.edu
[3] Department of Geological Sciences, University of Florida, Gainesville, FL 32608, USA; kamenov@ufl.edu
* Correspondence: JESQUIVEL@dental.ufl.edu

Received: 12 September 2020; Accepted: 2 October 2020; Published: date

(1) All values are the Intensity reading in cps (counts per second)

(2) Data acquired in Medium Resolution (MR), Rh and Re used as internal standards for correlation.

(3) All samples from soaking experiment are diluted 25× using Diluent Acid. Appropriate multiplication must be done to convert the raw readings to ppb reported in manuscript

Table S1. Titanium Calibration Intensity Avg.

Isotope	Blank	STD4	STD3	STD2	STD1
Ti49(MR)	23	46.7	382.2	3558.2	34675.5
Cu63(MR)	26.9	555.7	3401.4	34157.2	331376.7
Rh103(MR)	58.4	346463.8	341935.1	344990	339252
Ag107(MR)	78.7	207.4	1667	16113.9	156421.6
Sn118(MR)	12	213	2218.6	22369	222231.1
Re185(MR)	35.6	193608.3	190063	192073.2	193937.2
Hg202(MR)	136.1	188	193.5	279.6	441.7

Table S2. Blank Test Solutions Used in Experiment Intensity Avg.

Isotope	Diluent Acid	pH 2 Buffer	pH 7 Buffer	pH 10 Buffer
Ti49(MR)	29.6	45.9	511.9	328.2
Cu63(MR)	96.3	113	104.6	1910.8
Rh103(MR)	353049.8	354796.4	366629.4	368963.8
Ag107(MR)	79.6	39.8	25	22.2
Sn118(MR)	6.5	87	293.5	90.7
Re185(MR)	197853.4	198151	200808.6	194522.4
Hg202(MR)	221.3	380.6	435.2	608.5

Table S3. Solutions from Soaking Ti Rods ONLY (no amalgam) Intensity Avg

Isotope	pH 2	pH 7	pH 10
Ti49(MR)	51.9	508.2	5459.2
Cu63(MR)	185966711.6	32456.9	60331299.7

Rh103(MR)	378170.9	367666.5	373206.7
Ag107(MR)	6145.6	1250.9	12654.8
Sn118(MR)	40052.9	74034.3	40192999
Re185(MR)	204491.3	192682	198040.4
Hg202(MR)	2442.9	499.2	51086090.9

Table S4. Solutions from Soaking Ti Rods with amalgam Intensity Avg

Isotope	pH 2	pH 7	pH 10
Ti49(MR)	27.4	540.1	4792.4
Cu63(MR)	240195924	21866.7	33620312.8
Rh103(MR)	381132.2	374073.9	374382.8
Ag107(MR)	3832.5	64.8	17426.6
Sn118(MR)	18737.1	35895	17536470.5
Re185(MR)	205655.6	201670	198003.8
Hg202(MR)	1407.5	511.2	17709765.7

Table S5. Final Wash of ICP-MS to demonstrate memory effect of Hg

Isotope	5% HNO_3 Wash
Ti49(MR)	21.5
Cu63(MR)	1496.5
Rh103(MR)	48.9
Ag107(MR)	20.4
Sn118(MR)	1079.4
Re185(MR)	25.2
Hg202(MR)	359544.4

© 2020 by the authors. Submitted for possible open access publication under the terms and conditions of the Creative Commons Attribution (CC BY) license (http://creativecommons.org/licenses/by/4.0/).
